# A New Framework for Performing Cardiac Strain Analysis from Cine MRI Imaging in Mice

**DOI:** 10.1038/s41598-020-64206-x

**Published:** 2020-05-07

**Authors:** K. Hammouda, F. Khalifa, H. Abdeltawab, A. Elnakib, G. A. Giridharan, M. Zhu, C. K. Ng, S. Dassanayaka, M. Kong, H. E. Darwish, T. M. A. Mohamed, S. P. Jones, A. El-Baz

**Affiliations:** 1 BioImaging Laboratory, Department of Bioengineering, University of Louisville, Louisville, KY USA; 2 Electronics and Communications Engineering Department, Faculty of Engineeering, Mansoura University, Mansoura, Egypt; 3 Department of Radiology, Department of Medicine, University of Louisville, Louisville, KY USA; 4 Diabetes and Obesity Center, Department of Medicine, University of Louisville, Louisville, KY USA; 5 Department of Bioinformatics and Biostatistics, SPHIS, University of Louisville, Louisville, KY USA; 6 Mathematics Department, Faculty of Science, Mansoura University, Mansoura, Egypt; 7 Division of Cardiovascular Medicine, Department of Medicine, University of Louisville, Louisville, KY USA

**Keywords:** Cardiology, Medical research

## Abstract

Cardiac magnetic resonance (MR) imaging is one of the most rigorous form of imaging to assess cardiac function *in vivo*. Strain analysis allows comprehensive assessment of diastolic myocardial function, which is not indicated by measuring systolic functional parameters using with a normal cine imaging module. Due to the small heart size in mice, it is not possible to perform proper tagged imaging to assess strain. Here, we developed a novel deep learning approach for automated quantification of strain from cardiac cine MR images. Our framework starts by an accurate localization of the LV blood pool center-point using a fully convolutional neural network (FCN) architecture. Then, a region of interest (ROI) that contains the LV is extracted from all heart sections. The extracted ROIs are used for the segmentation of the LV cavity and myocardium via a novel FCN architecture. For strain analysis, we developed a Laplace-based approach to track the LV wall points by solving the Laplace equation between the LV contours of each two successive image frames over the cardiac cycle. Following tracking, the strain estimation is performed using the Lagrangian-based approach. This new automated system for strain analysis was validated by comparing the outcome of these analysis with the tagged MR images from the same mice. There were no significant differences between the strain data obtained from our algorithm using cine compared to tagged MR imaging. Furthermore, we demonstrated that our new algorithm can determine the strain differences between normal and diseased hearts.

## Introduction

Mice are the most frequently used species for *in vivo* modeling of cardiovascular diseases. The ability for genetic manipulation and established surgical models has made the mouse model a favorite to study the role of different genes in cardiovascular diseases^1^. Magnetic resonance imaging (MRI) is the most accurate imaging method (gold standard) to quantify cardiac function^2–6^. The major advantages of MRI are the clear tissue contrast, ability to rebuild 3D structure and function, and high reproducibility^7^. Currently, cardiac MRI is clinically used to assess heart structure, myocardial function, viability, and perfusion in humans^8^. Various mouse studies by our group and others^2,9–11^, used standard cine cardiac MRI to assess cardiac structure and functional parameters, e.g., end diastolic volume (EDV), end systolic volume (ESV), and ejection fraction (EF). However, strain analysis of myocardial contractility requires tagged cardiac MRI, which is time consuming and hard to interpret, especially in mice. This difficulty is due to the small size of the mouse heart, which does not allow the formation of multiple 1*mm*^2^ diamonds within the ventricular wall for strain analysis. Therefore, there is a lack of studies on assessing strain analysis from MRI imaging. Strain analysis is essential in completing the picture regarding the cardiac function as it is the only parameter that can differentiate between active and passive movement of myocardial segments, quantify intraventricular dyssynchrony and evaluate longitudinal myocardial shortening, that are not visually assessable. In addition, measuring myocardial strain enables comprehensive assessment of diastolic myocardial function, which is not reflected in EF^12^.

Deep learning techniques, in particular convolutional neural networks (CNN)^13^, have been successfully implemented in medical imaging applications that range from traditional image processing tasks such as segmentation and registration to the implementation of advanced computer aided diagnostic systems for disease classification^14,15^. The fully convolutional neural network (FCN) is a deep learning model built primarily from convolutional layers and can be trained to perform object segmentation or even semantic segmentation for the processed image^16^. Various implementations of FCNs have been presented in literature for the automated segmentation and quantification of the LV from human cardiac cine images^17–20^. These approaches provided accurate segmentation and quantification of the LV global indices. However, there has been very little research in the area of deep learning applied to mice cardiac MRI data^21^.

The primary objective of this manuscript is to develop an automated pipeline for accurate estimation of strain from standard cine MRI in mice for both sham and myocardial infarction (MI) subjects, to obviate the need for tagged imaging in mice. Since myocardial segmentation is a crucial first step in processing and analyzing left ventricle (LV) functional indices, a deep learning-based approach for automatic segmentation of myocardium borders was developed. Subsequently, a Laplace-based approach to track myocardial points through the cardiac cycle was developed to accurately assess the strain from the standard cine cardiac MRI. The Laplace-based method was validated by comparing it to the strain analysis outcome obtained by the tagged imaging which is currently the gold standard for strain analysis. Finally, we also used our pipeline to estimate other global and local cardiac indices.

## Results

**Figure 1:**
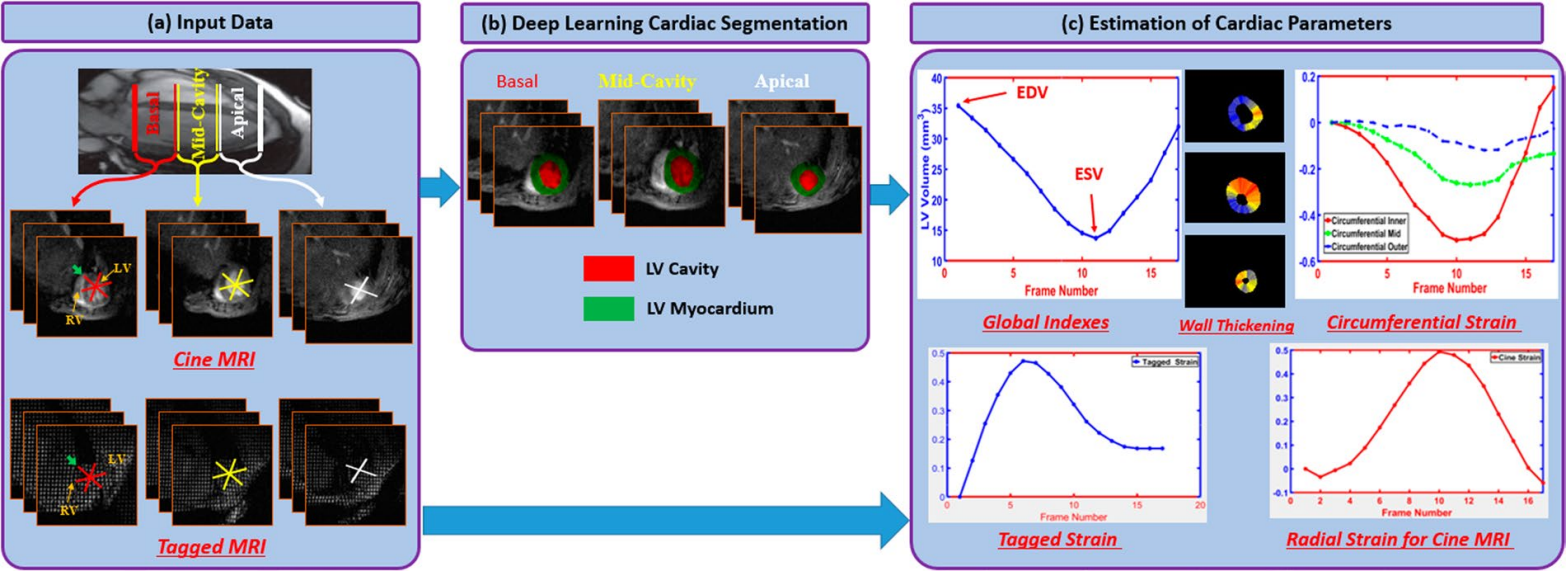
The proposed framework for the analysis of cardiac MRI data.

Our proposed framework for the automated quantification of LV functional indices is composed of two primary stages: A deep learning-based segmentation of the LV cavity and myocardium using a FCN followed by the estimation of functional and structural cardiac parameters. The latter includes global volumetric indices (e.g., EDV, ESV, EF) and local indices including wall thickening and myocardium strain. The overall framework is depicted in Fig. 1, and its development and validation will be discussed in more detail in the next subsections.

### Development of an automated algorithm for myocardial segmentation

**Figure 2:**
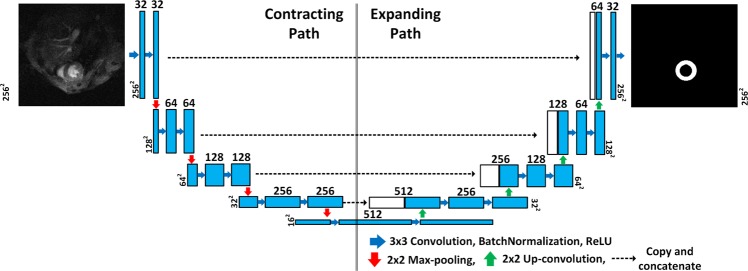
The architecture of the used U-net for LV segmentation.

Deep learning-based segmentation outperformed traditional image segmentation techniques. In this study, we used an FCN similar to the U-net which was designed for achieving medical image segmentation and presented in MICCAI 2015^22^. The architecture of the used network is shown in Fig. 2, where the input 2D image is passed through a contracting path followed by an expanding path. In the contracting path, the image is processed by successive blocks of convolution, rectified linear unit (ReLU) activation, and max pooling operations. This processing reduces the spatial dimensions and produces an abstract representation for the input in a layer called bottleneck. The spatial dimension is restored in the expanding path by applying up-convolution. Up-convolution operation increases the resolution of the input by a deconvolution filter that can be learned during network training^16^. Furthermore, the network has skip connections that copy and concatenates high resolution feature maps from the contracting path to the expanding path. This result in fine segmentation. The network is trained by pairs of cardiac MRI 2D images, *X*, and their corresponding binary ground truth LV manual annotations, *Y*. The output of the network to an image *x* is a one layer segmentation map *ŷ*, where each pixel contains a probability of belonging to the segmented object, which in this case is the LV myocardium. The map *ŷ* is obtained by employing a sigmoid layer as the final layer in the network. A loss function was then used to compare this map against the true binary image *y*.

Segmentation using FCNs is a dense classification in the image spatial domain. A major challenge is that the network tends to be biased toward the class that comprises the majority of pixels during training. This class-imbalance issue exists in our segmentation task because the LV pixels are only a small proportion compared to the surrounding tissues. Deep learning networks that depend on the conventional binary cross entropy (BCE) loss function are prone to class-imbalance^23^. Consequently, we propose a novel loss function that consists of two terms to address the class-imbalance. First, the conventional BCE loss described as follows:

1 $${L}_{BCE}=\mathop{\sum }\limits_{i\mathrm{=1}}^{N}-({y}_{oi}\,\log ({\hat{y}}_{oi})+{y}_{bi}\,\log ({\hat{y}}_{bi}))$$

where *N* is the number of the pixels, and *y*_*oi*_, and *y*_*bi*_ are the true labels for pixel *i* obtained from ground truth *y* (*y*_*oi*_ is 1 when *i* lies in the object pixel and 0 for the background, and vice versa with *y*_*oi*_). *ŷ*_*oi*_ and *ŷ*_*bi*_ are the predicted probabilities that a pixel *i* in the object and the background surrounding tissues, respectively. Second, a loss term that combines the sensitivity and specificity is defined as follows:

2 $$\begin{array}{l}{L}_{ss}=2-(Sensitivity+Specificity)=2-(\frac{\mathop{\sum }\limits_{i\mathrm{=1}}^{N}{\hat{y}}_{oi}{y}_{oi}}{\mathop{\sum }\limits_{i\mathrm{=1}}^{N}{\hat{y}}_{oi}{y}_{oi}+\mathop{\sum }\limits_{i\mathrm{=1}}^{N}{\hat{y}}_{bi}{y}_{oi}}+\frac{\mathop{\sum }\limits_{i\mathrm{=1}}^{N}{\hat{y}}_{bi}{y}_{bi}}{\mathop{\sum }\limits_{i\mathrm{=1}}^{N}{\hat{y}}_{bi}{y}_{bi}+\mathop{\sum }\limits_{i\mathrm{=1}}^{N}{\hat{y}}_{oi}{y}_{bi}})\end{array}$$

By definition, high sensitivity and the specificity requires minimization of false negatives (FNs) and false positives (FPs). The final loss function *L* was the sum of the two terms as follows:

3 $$L=\alpha {L}_{BCE}+\beta {L}_{ss}$$

where the hyper-parameters *α* and *β* control the assigned weights for each term in the equation. In our formulation, *α* + *β* = 1, and a higher value was assigned to *β* compared to *α*, to reduce the effect of BCE to account for the class imbalance while assigning a higher weight for the minimization of the sum of FPs and FNs. A grid search approach was adopted to determine the optimal values of *α* and *β* when the value of the Dice similarity coefficient (DSC) was the optimization criterion. The searched values for *α* and *β* were in the range of 0:0.1:1 with *α* + *β* = 1 constraint. Tversky loss has been proposed by Salehi *et al*.^24^ to improve deep learning segmentation in the presence of a class-imbalance problem. To establish the potential of the proposed loss function, its performance was compared with the existing methods.

For a test image x^*^, the network output was the underlying predicted segmentation map ŷ^*^, where each pixel was assigned a probability by the sigmoid layer. To obtain the binary segmentation, Otsu thresholding^25^ was applied on ŷ^*^. Further post-processing operations were applied on the binary segmentation: (i) connected component with the maximum number of pixels was kept and other FP components were removed (ii) morphological gap filling to fill the gaps in the final binary segmentation. After post processing, we can infer the segmentation quality of our network for both LV cavity and myocardium.

The proposed FCN network was trained and tested using cine MRI images of six mice in a leave-one-out cross-validation scheme where six iterations of consecutive training and testing were performed. In each iteration, the 2D images of five cases (approximately 750 images) were used for training the network and the images of one case (approximately 150 images) were used for testing the network, where the averages of the segmentation metrics were estimated along the tested images. This process was repeated six times and the final averages of the segmentation metrics were estimated, and these values were reported to evaluate our segmentation technique. The images were re-scaled to spatial dimensions of 256 × 256 pixels to maintain consistent dimensions through our network. A data augmentation strategy that depends on random translation, scaling, and rotation was adopted. The Kaiming initialization^26^ was used as a weight initializer for the convolutional layers. The Adam optimizer^27^ with a learning rate of 0.001 and a learning momentum of 0.9 was used for network optimization. We used 100 epochs and a stop criterion of 5 waiting iterations. To perform early stop, we used the loss value of a validation set that consists of 20% of the training set. Hence, our model performs an early stop before the end of 100 epochs, if the validation loss did not decrease for 5 consecutive epochs. Finally, the best model with the lowest loss value on the validation set was used for testing. The Pytorch software was used to implement, train, and test our network.

### Algorithm for calculating cardiac function and structure parameters

After the segmentation of the LV myocardial border, physiological heart parameters (both local and global) can be estimated for cardiac functional assessment. The estimation of the local and global ventriculometrics requires accurate localization of the inner and outer wall boundaries, especially for local ventriculometrics such as strain, which require tracking of myocardium contour points. Wall thickness calculation requires only accurate co-allocation of the corresponding point on the heart wall. Thus, the critical step in analysis is the accurate localization of the myocardial points. To avoid issues associated with intensity variations between subjects’ scans and the lack of strong edges, especially on the outer boundary, an approach that exploits geometric features rather than image intensities was developed. This process co-allocates the corresponding border pairs or tracks myocardial points through the cardiac cycle. A geometric method was applied to determine geometric features, and the matching of myocardial points through multiple time frames by solving the Laplace equation between each two successive contours^28–30^:

4 $${\nabla }^{2}\gamma =\frac{{\partial }^{2}\gamma }{\partial {x}^{2}}+\frac{{\partial }^{2}\gamma }{\partial {y}^{2}}=0$$

where *γ*(*x*, *y*) is the estimated electric field between the inner and outer wall borders. Generally, the solution of Eq. (4) resulted in intermediate equipotential surfaces and streamlines, being everywhere orthogonal to all equipotential surfaces and establishes natural pixel correspondences between the boundaries. In order to estimate *γ*(*x*,*y*), a second-order central difference method and the iterative Jacobi approach were used as given by:

5 $${\gamma }^{i+1}(x,y)=\frac{1}{4}\{{\gamma }^{i}(x+\Delta x,y)+{\gamma }^{i}(x-\Delta x,y)+{\gamma }^{i}(x,y+\Delta y)+{\gamma }^{i}(x,y-\Delta y)\}$$

where *γ*^*i*^(*x*, *y*) is the estimated electric field at (*x*, *y*) during the *i*^*th*^ iteration; and Δ*x* and Δ*y* are the step length or resolution in *x* and *y* directions, respectively. Algorithm 1 summarizes basic steps for the co-allocation of pixel-wise correspondences using the Laplace equation.

**Figure:**
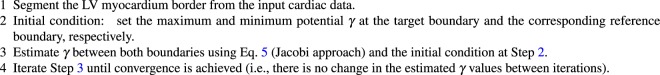
Algorithm 1 Solution of the Laplace equation between wall borders (tracking over the time series).

#### Algorithm for calculating myocardial strain from cine MR images

Accurate strain estimation plays an important role in the early detection of many cardiac diseases including coronary atherosclerosis. Traditionally, myocardial strain is estimated from tagged MRI data, which is usually acquired for the same subject during cine MRI acquisition. However, due to the changes in the acquisition protocols, we propose to estimate strain using cine MRI from mice. Namely, we will use a single modality for complete analysis of a subject to avoid problems such as an unequal number of slices, different slice locations, and different number of frames per slice. Circumferential and radial strain were estimated.

**Figure 3:**
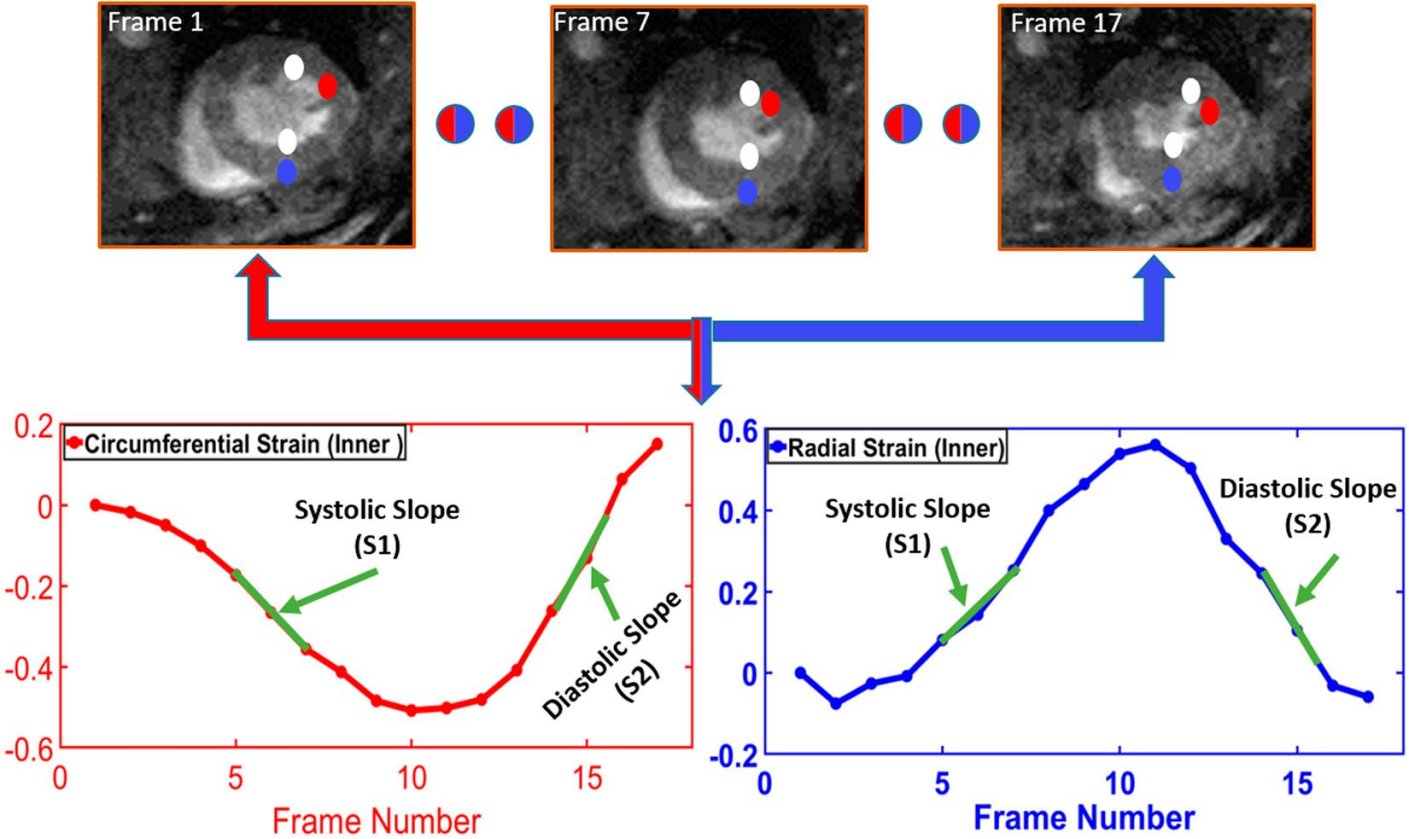
Illustration of the tracking process throughout the cardiac cycle to estimate the radial and circumferential strains, as well as two functional metrics systolic slope (S1) and diastolic slope (S2) on circumferential and radial strain.

Strain estimation from cine images depends mainly on tracking the LV wall geometry. In this paper, we developed a Laplace-based approach to track the LV wall points by solving the Laplace equation between the LV contours of each two successive image frames over the cardiac cycle (see Algorithm 1). Following tracking, the strain estimation was performed using a Lagrangian-based approach. The Lagrangian strain calculation for finitely small displacement was used to estimate the strain^31^:

6 $${\varepsilon }_{L}=(\begin{array}{cc}{\varepsilon }_{{x}_{1}} & {\varepsilon }_{{x}_{1}{x}_{2}}\\ {\varepsilon }_{{x}_{2}{x}_{1}} & {\varepsilon }_{{x}_{2}}\end{array})$$

where ${\varepsilon }_{{x}_{1}}$ and ${\varepsilon }_{{x}_{2}}$ are the normal strain components; also ${\varepsilon }_{{x}_{1}{x}_{2}}$ and ${\varepsilon }_{{x}_{2}{x}_{1}}$ are the shear strain components. According to our data in Fig. 3, the strain cycle is complete within the 17 frames of the cine and tagged images. therefore, we focused all our analysis on frames 1–17 of the cardiac cycle. Algorithm 2 summarizes basic steps for calculating myocardial strain for both radial and circumferential directions.

**Figure:**
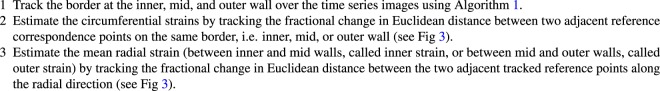
Algorithm 2 Basic steps for calculating the circumferential and radial strain.

#### Algorithm for calculating myocardial strain from tagged MR images

As the standard way of strain analysis, we also estimated the strain from tagged MRI. Myocardial tagging is an MRI technique specialized in the assessment of the heart’s contractile function. In this technique, the heart motion is imaged by creating a spatial pattern of saturated magnetization in the wall of the heart at a certain time, followed by capturing the deformation of this pattern during the cardiac cycle. Tagged MRI is more suited for cardiac strain analysis than conventional MRI that lacks the well identifiable landmarks within the heart wall. However, the mice tagged MRI data has a very poor resolution and very low signal-to-noise ratio compared to the human cardiac MRI, which creates difficulty in using commercially available software to obtain reliable strain measurements. Thus, we developed a tracking method to estimate the myocardial motion during the cardiac cycle. Myocardial motion provides valuable information for cardiac strain assessment. The tracking points of tagged MRI were identified by determining the maximum correlation between a window of size 3 × 3 and a window of size 5 × 5 for pixel (*i,j*) in frame number *n* and frame number *n* + 1, respectively, as illustrated in Fig. 1 in the supplementary materials. Also, Algorithm 3 summarizes the basic steps for estimating myocardial strain for tagged MRI.

**Figure:**
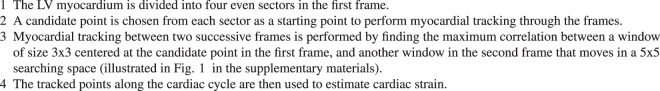
Algorithm 3 Strain estimation using tagged MRI.

#### Wall thickness and thickening

In addition to local strain estimation, the wall thickness during systole and diastole was estimated. Systole results in an increase in LV wall thickness. Distances between corresponding points on the inner and outer wall borders were used to determine the wall thickness and wall thickening. The co-localization of the corresponding points on the heart wall is estimated from the calculated field vectors by solving the Laplace equation between the segmented heart borders (see Algorithm 1).

**Figure 4:**
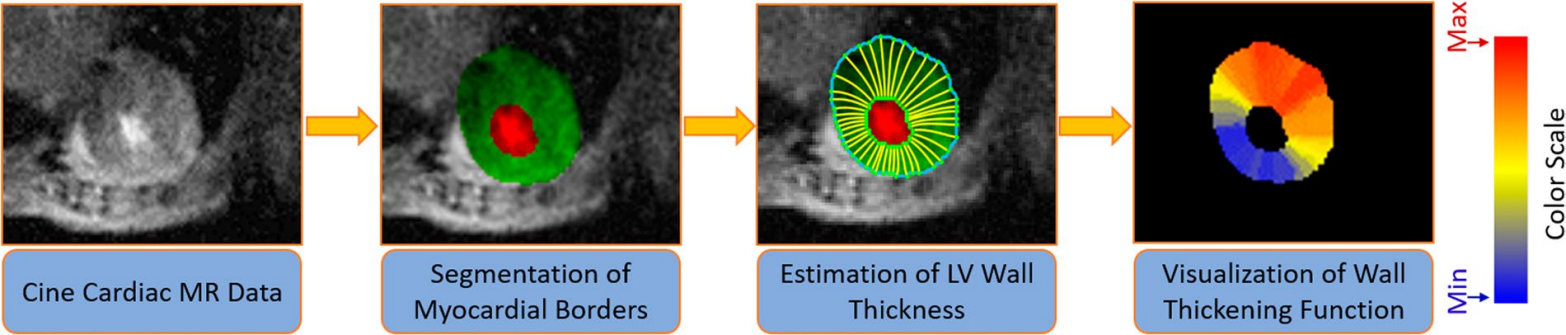
The sequential steps for wall thickening analysis and visualization using parametric maps.

**Figure 5:**
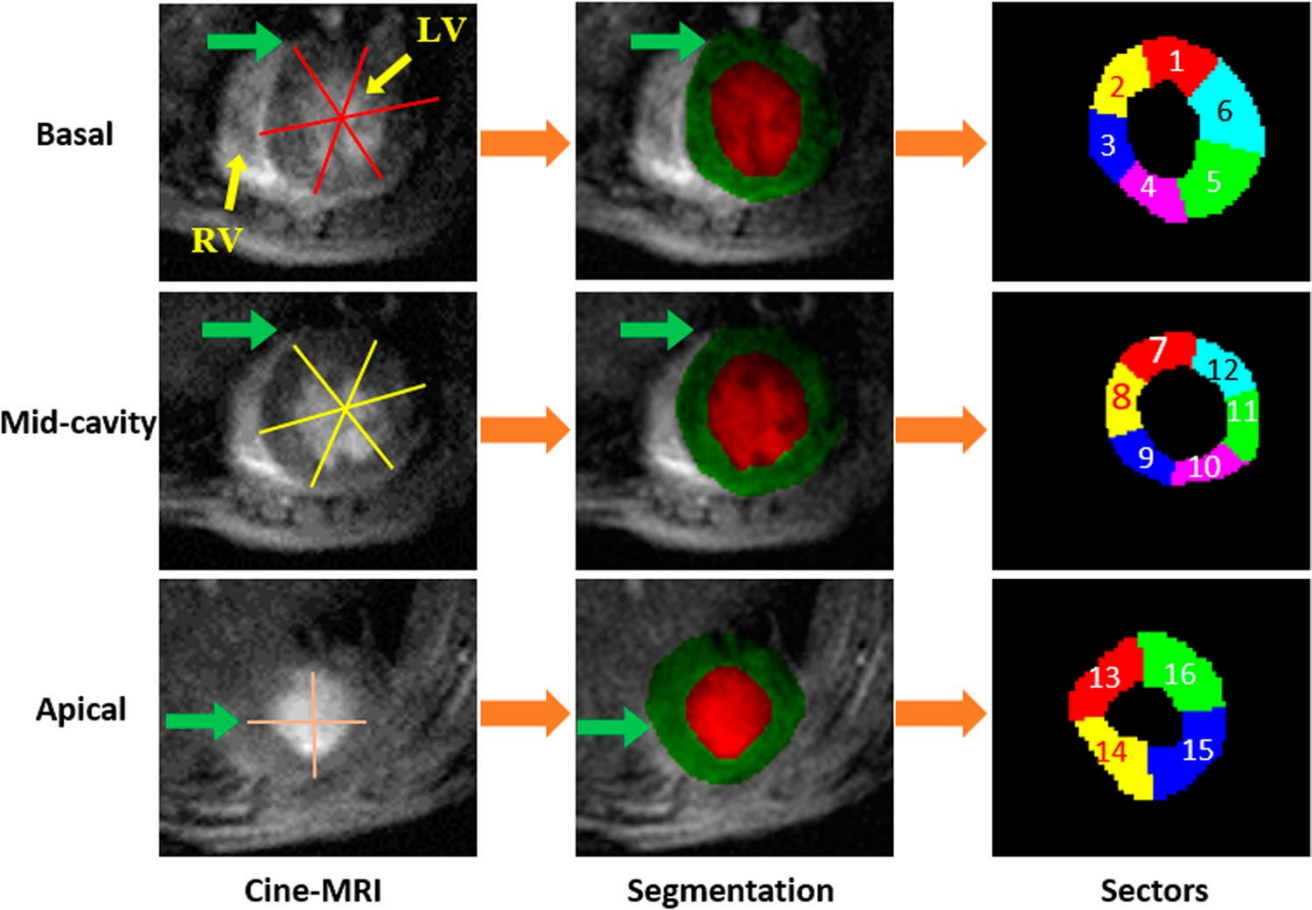
Illustration of the 17 segments model^35^ for one subject. Section division starts counterclockwise from the green arrows.

After thickening estimation, a color-coded parametric map is used for visualization. A schematic illustration for the thickening estimation and visualization is shown Fig. 4. In addition to parametric maps, sectors analysis for wall thickness was conducted using a seventeen-segment model. According to this model, the heart is divided into six segments at the basal and mid-cavity levels and into four segments at the apical level. The last segment is at the tip of the heart, and hence is not used in the analysis (see Fig. 5. The purpose of this analysis is to investigate changes in myocardial function.

#### Global Ventriculometrics

In addition to the local ventriculometrics, the evaluation of heart function relies primarily on global volumetric indices, such as EDV, ESV, and EF. Our methodology aims to provide an automatic and accurate way to evaluate global heart function. Thus, we estimate parameters related to the LV function and mass^32^, which are defined as follows:

1. Left ventricular volume (LVV): the preliminary measure that is required for the derivation of other important parameters such as EF. Simpson’s method^33^, which computes LVV as the sum of several smaller volumes with the same configuration, was used.
2. Left ventricular mass (LVM): used to characterize LV hypertrophy. To estimate the LVM, two assumptions were made: i) the interventricular septum is part of the LV and ii) the volume of the myocardium, *V*_*m*_, is the total volume delineated by the epi-cardial contours of the LV, *V*_*EpC*_, minus the LV cavity volume, *V*_*EnC*_ at the phase of end diastolic (ED). The LVM can be estimated by multiplying *V*_*m*_ by the density of the myocardial tissue *ρ* = 1.05*g*/*cm*^3^:
  7 $${V}_{m}={V}_{EpC}({t}_{ED})-{V}_{EnC}({t}_{ED})$$
  8 $$LVM=\rho \times {V}_{m}$$
  For inter-subject comparisons, the LVM was normalized to the body weight or total surface area.
3. Stroke volume (SV): the blood volume ejected between the phase of ED and the phase of end systolic (ES).
  9 $$SV={V}_{EnC}({t}_{ED})-{V}_{EnC}({t}_{ES})$$
  SV can be normalized to the total body surface area to obtain the SV index (SVI).
4. Ejection fraction (EF): is another commonly used parameters for the evaluation of LV function. It is defined as follows:

10 $$EF=\frac{SV}{{V}_{EnC}({t}_{ED})}\times \mathrm{100 \% }$$

### Validation of the functional algorithm in detecting changes in cardiac function following injury

The primary goal of this manuscript is to demonstrate the ability of the cine MRI to estimate myocardial strain without using tagged MRI for mice. Moreover, the proposed method will enable an accurate estimate of the correlation coefficients between the strain index and other performance indices derived from cine images, including global (e.g., EF) and local (e.g., wall thickening) indices.

#### Myocardial segmentation

**Figure 6:**
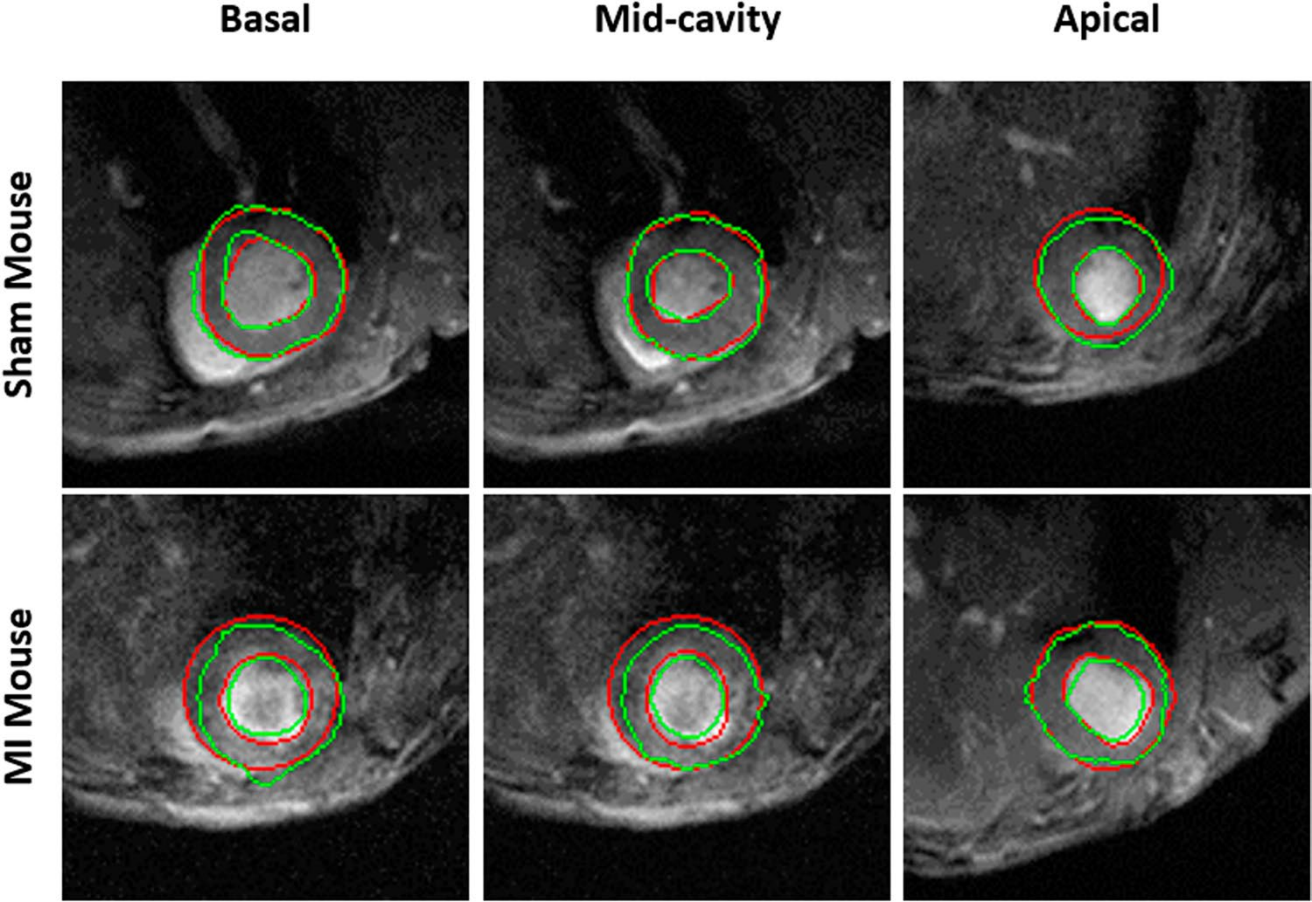
Sample segmentation results for sham and myocardial infarction (MI) subjects at different cross-sections of the heart. The segmentation illustrates the cavity and the myocardium obtained using our framework (green) and ground truth (red).

The first step and a crucial part in the analysis of cardiac data is myocardial segmentation. Myocardial border segmentation was performed using the proposed deep learning framework presented in Section 2.1. The ground truth segmentation was obtained by manual delineation of the LV wall borders by T. M. A. Mohamed, a contributing co-author. Sample results of the proposed segmentation approach are illustrated in Fig. 6, where the LV cavity is shown in red and the myocardium is shown in green overlaid on the gray image.

To evaluate the accuracy of our segmentation pipeline, we used the DSC to characterize the spatial overlap between segmented and ground truth segmentation for each LV slice, as well as the Hausdorff distance (HD) to characterize the closeness of the segmented contours to their ground truth counterparts for each LV slice. The overall segmentation accuracy for LV cavity and myocardium with respect to the gold standard is summarized in Table 1 for the proposed and other loss functions. Table 1 shows the average segmentation accuracies per LV slice. The best segmentation performance from our loss was obtained at *α* = 0.2 and *β* = 0.8.

**Table 1:** Comparison between the segmentation accuracy of our framework with the proposed loss and other methods using both DSC and HD metrics.

	BCE Loss		Proposed Loss		Tversky Loss	
	Cavity	Myocardium	Cavity	Myocardium	Cavity	Myocardium
**DSC** (%)	90.02 ± 2.11	87.33 ± 2.22	96.45 ± 1.56	93.26 ± 2.21	94.55 ± 1.41	92.32 ± 1.74
**HD** (mm)	0.91 ± 0.16	1.23 ± 0.14	0.59 ± 0.05	0.87 ± 0.15	0.63 ± 0.07	0.91 ± 0.13

The metrics are presented in the form of mean±SD for the LV cavity and myocardium of the heart.

#### Global ventriculometrics

**Figure 7:**
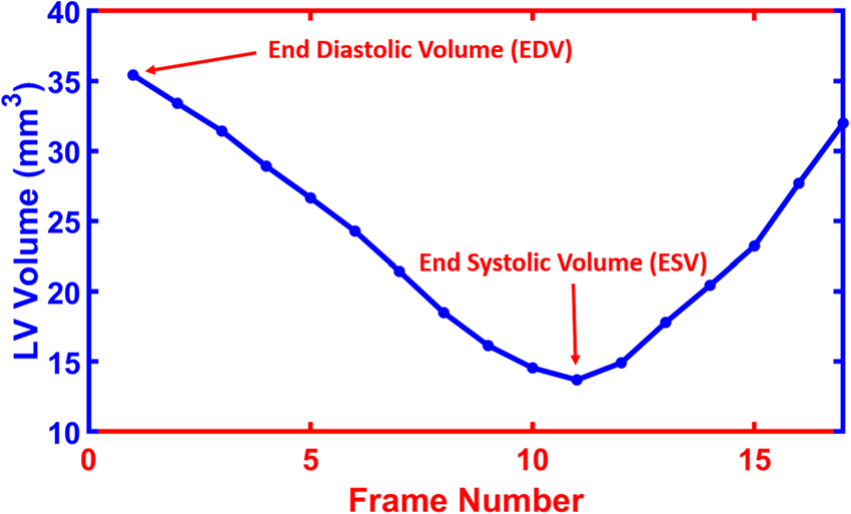
LV curve shows the cavity volume along the cardiac cycle and the time frames used to estimate the EF.

**Figure 8:**
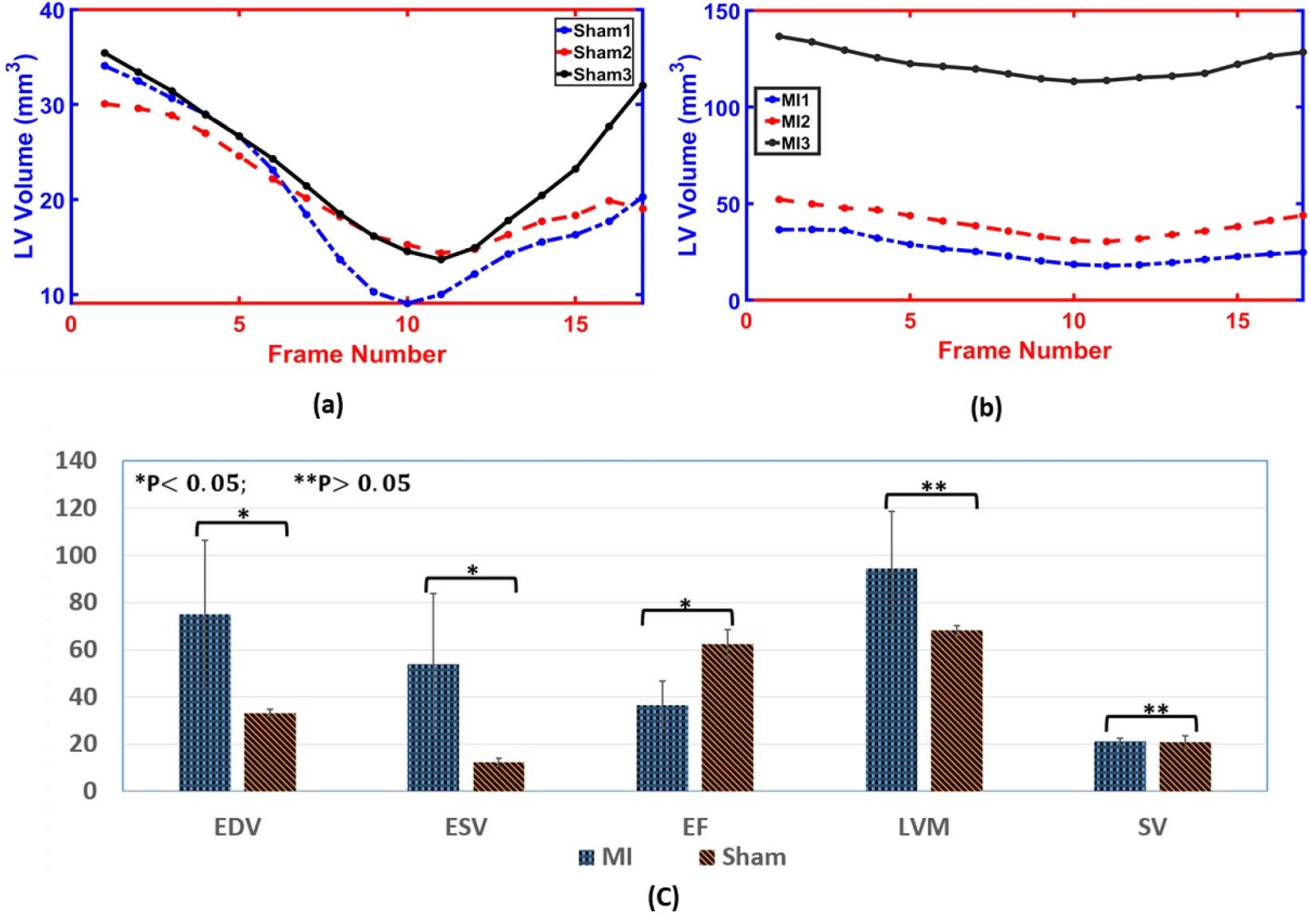
Global functional indices for both sham and myocardial infarction (MI) subjects: (**a**) LV ventricular curves for sham groups, (**b**) LV ventricular curves for MI groups (each curve represents the summation of the cavity areas over the entire physiological cardiac cycle); and (**c**) a bar plot of the average of all subjects for functional indices (EDV, ESV, EF, LVM, and SV) and their standard error for mean (SEM) bar, as well as statistical t-test for comparison.

Functional indices can help accurately quantify cardiac function, detect local, and global cardiac diseases. Some indices like EDV, ESV, EF, LVM, and SV are commonly used global assessments of the heart. The LV volume curves are constructed at any point of time during the cardiac cycle. Relevant parameters of the LV can be derived from those curves. The EDV and ESV are extracted from LV curves, as illustrated in Fig. 7. The LVM, SV, and EF are computed using Eqs. (8–10), respectively. The curves for all MI and sham subjects are shown in Fig. 8(a,b), respectively. In addition, the results for all functional indices (EDV, ESV, EF, LVM, and SV) are estimated and summarized as a bar plot of the average of all subjects for these indices and their standard error for mean (SEM) bar (see Fig. 8(c)).

The comparison between each functional index for sham and MI groups were carried out using the two-sample Student’s t-test (see. Figure 8(c)). In the animal experiments, three mice per group was utilized. This sample size still enables detection of an effect size of 3.1 with power 80% at a significance level 0.05. Log transformation was applied due to the large standard deviations between the two groups. The test illustrated that there was a statistically significant difference (P-value < 0.05) in EDV, ESV, and EF between sham and MI groups. The sham group had higher EF compared to the MI group. EDV and ESV values for the MI group were much higher compared to the sham group. On the other hand, LVM and SV have no statistically significant difference (P-value > 0.05) between sham and MI groups. For SV, the average value for MI and the sham group were approximately equal.

#### Wall thickening

Figure 4 shows the estimation of the LV wall thickness from the point-to-point correspondences on the segmented cine images. For visualization assessment of the wall thickening, a pixel-wise parametric (color-coded) map was used. The representation example of the wall thickening color-map depiction is shown in Fig. 2 (supplementary). To preserve consistency and reduce the effect of noisy estimates of the wall thickening values, we employed a continuity analysis using the maximum a posterior (MAP) estimates for a 3-D generalized Gauss-Markov random field (GGMRF) probabilistic model^34^ for smoothing. In addition to parametric-maps visualization, local wall thickening can be estimated in each of the 17 segments of the heart^35^. The measurements of the 17 segments of the LV myocardium is presented in Table 2 for sham and MI groups. Additional explanation about the 17 segments is provided in Fig. 5.

**Table 2:** Wall thickening measurements for all subjects using the standardized myocardial 17-segments model^35^.

	Normalized wall thickening															
Section Number	1	2	3	4	5	6	7	8	9	10	11	12	13	14	15	16
MI mouse1	0.58	0.58	0.58	0.58	0.58	0.58	0.44	0.25	0.36	0.43	0.38	0.46	0.27	0.27	0.27	0.27
MI mouse2	0.27	0.27	0.27	0.27	0.27	0.27	0.46	0.39	0.24	0.17	0.08	0.23	0.14	0.14	0.14	0.14
MI mouse3	0.63	0.43	0.21	0.26	0.41	0.64	0.15	0.14	0.22	0.26	0.20	0.25	0.16	0.16	0.16	0.16
Sham mouse1	0.63	0.43	0.21	0.26	0.41	0.64	0.15	0.14	0.22	0.26	0.20	0.25	0.16	0.16	0.16	0.16
Sham mouse2	0.35	0.35	0.35	0.35	0.35	0.35	0.82	0.63	0.21	0.18	0.31	0.62	0.60	0.60	0.60	0.60
Sham mouse3	0.44	0.44	0.44	0.44	0.44	0.44	0.65	0.43	0.33	0.49	0.54	0.44	0.15	0.15	0.15	0.15

### Comparison between myocardial strain obtained from cine vs. tagged MRI images

**Figure 9:**
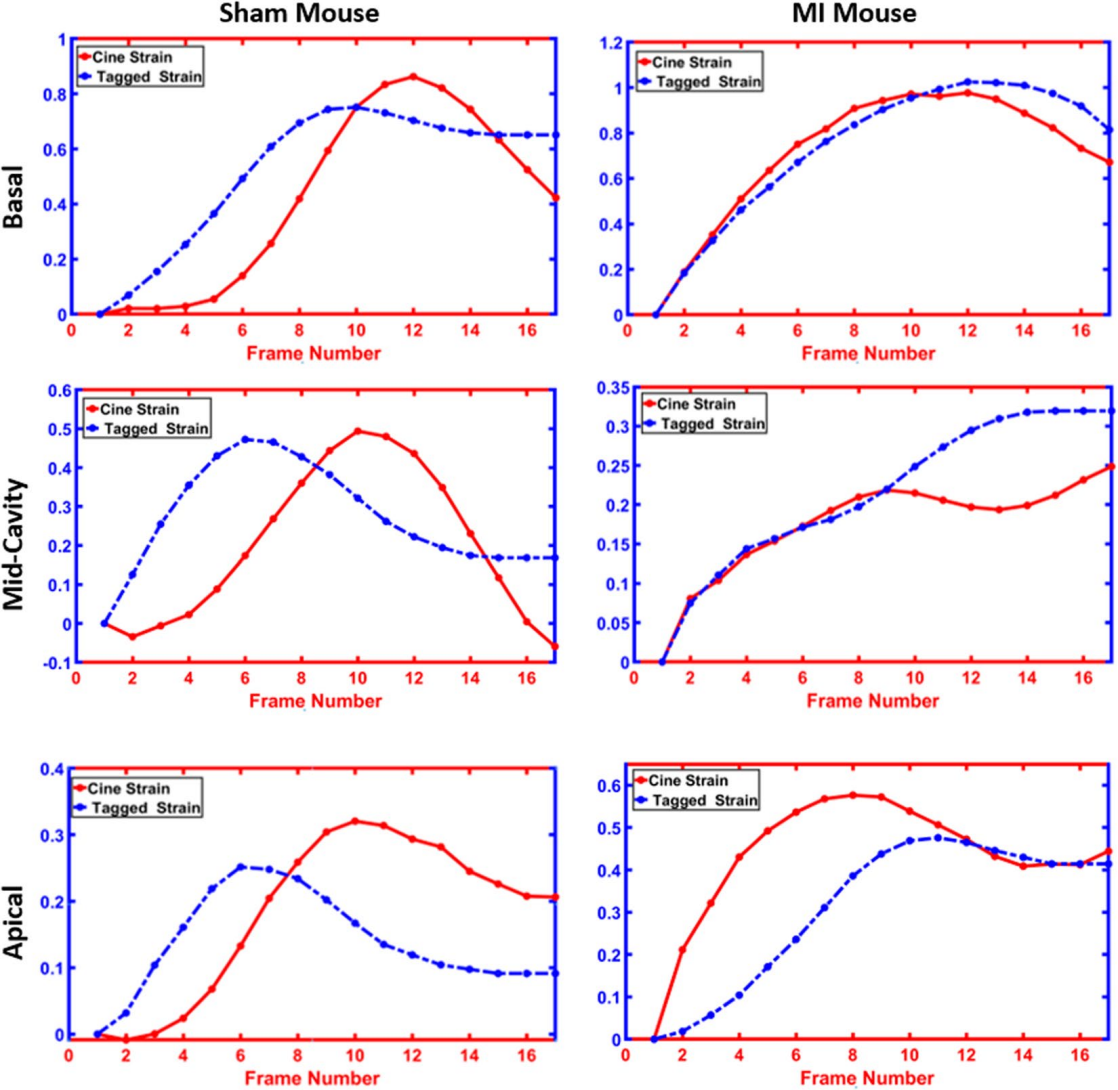
Average radial strain curves estimated from cine and tagged images at different cross-sections (rows) for all (**a**) sham and (**b**) myocardial infarction (MI) subjects.

Strain analysis provides critical insights about cardiac function. Our proposed method relies on a geometrical-based method to track the myocardial boundary and estimate functional strain for cine MRI and tagged strain. To estimate strain for cine images (both circumferential and radial strains) and construct strain curves, Algorithm 2 was applied. Figure 3 shows the tracking process throughout the cardiac cycle to estimate the radial and circumferential strains. Algorithm 3 was used to estimate strain for tagged images. Figure 9 demonstrates the average radial strain curves estimated from both cine and tagged images for each cross-section of the heart (i.e., basal, mid-cavity, apical) for both animal groups (i.e., sham and MI). As demonstrated in the figure, the cine and tagged strain curves have the same trend and good agreement. It is worth mentioning that, we are not showing the end of the cardiac cycle as we are showing up to frame 17 out of 22 frames. This is due to the occurrence of artifacts in the tagged images at the end of the cardiac cycle, which make it not reliable at this point. This is mainly due to the small size of the mouse heart and the incompatibility with the diamond structure of the tagged images.

In addition to the visual comparison, we used a statistical metric and the Bland-Altman analysis to assess curves agreements. First, the correlation coefficient (CorCoef) between the two curves and the mean squared error (MSE) of the strain curves were calculated. Table 3 shows the average of the CorCoef and MSE for comparison strain estimated from cine and tagged MRI for both sham and MI mice at different cross-sections. As demonstrated, there is a high CorCoef between the estimated strain from both modalities, which range between 0.737–0.966 for all sham and MI. On the other hand, the values of MSE are low and are in the range 0.024–0.08. As demonstrated in Fig. 9 strain curves for cine and tagged MRI have the same trend, which is also confirmed by the quantitative results in Table 3.

**Table 3:** Correlation coefficient (CorCoef) and mean squared error (MSE) for comparing strain estimated from cine (radial) and tagged MRI for both sham and MI.

	Basal		Mid-Cavity		Apical	
	CorCoef	MSE	CorCoef	MSE	CorCoef	MSE
**MI**	0.920	0.025	0.737	0.033	0.783	0.079
**Sham**	0.909	0.028	0.966	0.023	0.812	0.039

**Figure 10:**
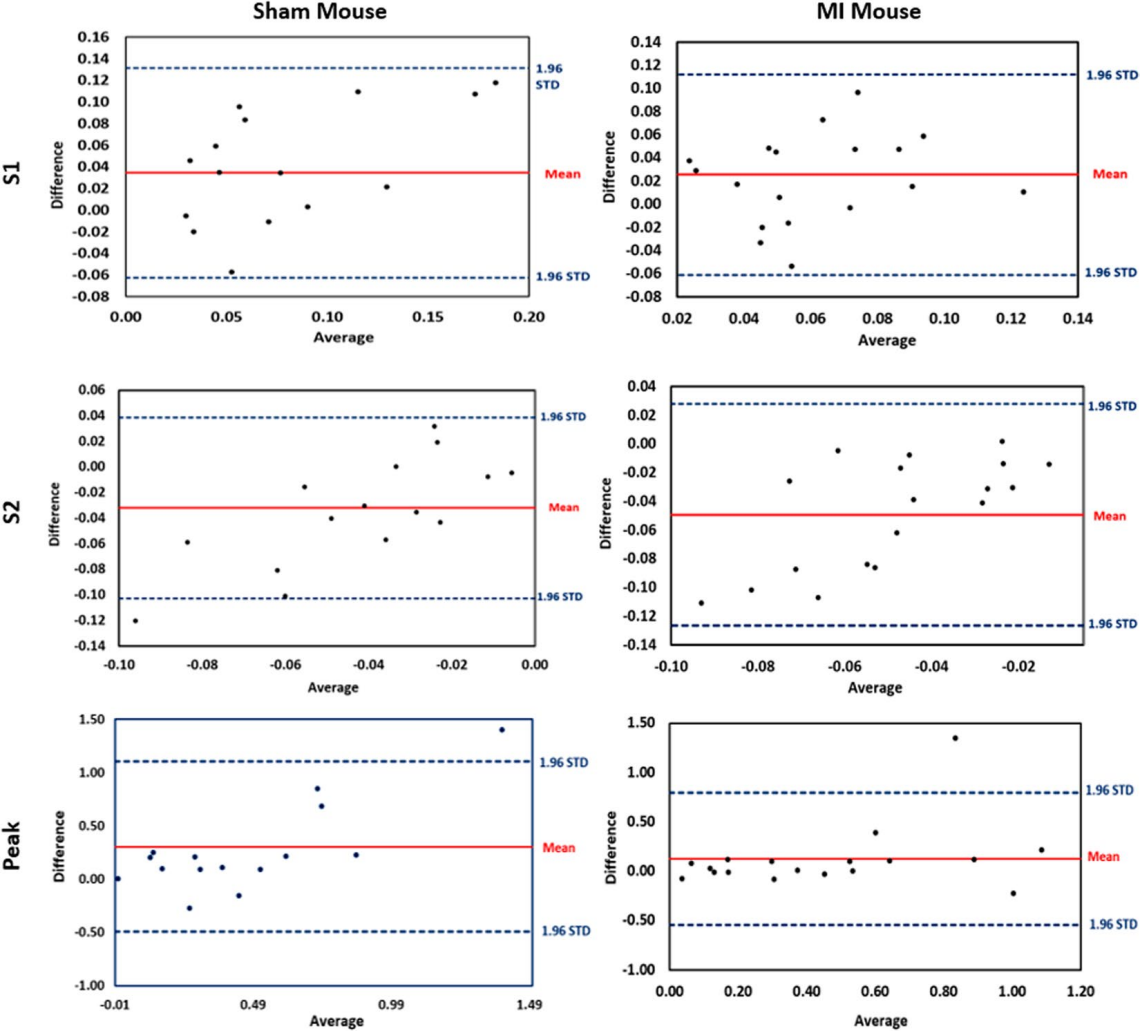
The Bland-Altman agreement plots for the slopes (S1 & S2) and the peak values of the estimated strain curves and the peak value for strain curves from cine and tagged images for all MI and sham subjects. Note that STD stands for standard deviation.

Besides statistical metric, the Bland-Altman analysis was constructed to validate the curves agreement. For evaluation, these plots show the bias (mean difference) and the 95% limits of agreement, standard deviation around the bias, between various metrics that are estimated from both cine and tagged imaging. Namely, the slopes of systolic (S1) and diastolic (S2) phases of the heart, and the peak strain value for the estimated strain curves (see Fig. 3). As shown in Fig. 10, the estimated metrics are in excellent agreement for both sham and MI subjects. In the Bland-Altman plot, the normality of the differences must be verified. Therefore, we applied the Shapiro-Francia test for normality with 5% significance level. the P-values for S1 (sham, MI), S2 (sham, MI), and peak (sham, MI) were (0.0914, 0.9767), (0.9434, 0.7446), and (0.0609, 0.1241), respectively. The P-values are larger than 0.05, so the test accepted normality. Also, data normality were accepted using Jarque-Berat, DAgostino Pearson, and Kolmogorov-Smirnov^36^ tests.

#### Circumferential strain for cine MRI

**Figure 11:**
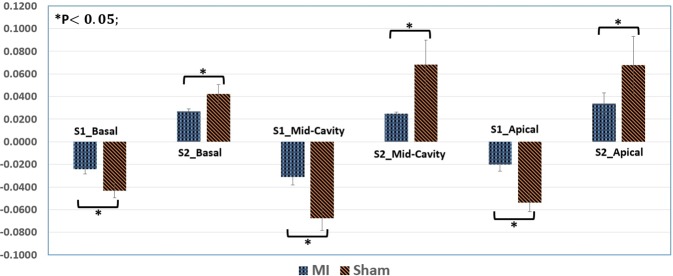
Bar plot for the mean and standard error for mean (SEM) of systolic (S1) and diastolic (S2) for circumferential strain that estimated for all three cross-sections (basal, mid-cavity, apical) to sham and myocardial infarction (MI) groups. The p-values were obtained based on the Student’s t-test.

To estimate the circumferential strain and construct strain curves, Algorithm 2 was applied. An example of the estimation circumferential strain for a sham and an MI subject is in the supplementary material (Fig. 3). This strain was estimated for all subjects (at different cross-sections of the heart) for the inner, mid and outer walls. As readily seen, there is a difference between the MI and sham groups. The MI case shows a reduced strain value especially at the outer wall. In addition, there is a difference in the circumferential strain measured at different locations in the myocardium. For example, the strain for the inner wall has a higher range compared with the mid and outer walls. In addition to visual comparison, the slope of quantitative analysis of the obtain strain curves was performed by estimating S1 and S2 for the circumferential strain curves as shown in Fig. 3 above. Figure 11 displays the bar plot for the mean and SEM of S1 and S2 for all three cross-sections (basal, mid-cavity, apical) to sham and MI groups.

In addition, the statistical analysis for each cross-section was performed using two sample Student’s t-test (see Fig. 11). Each of the basal and mid-cavity sections contain three slices, and apical includes two, which were averaged for each section for each subject. These data were used to examine the group difference between Sham and MI. Furthermore, log transformation was applied to make the measurement variable fit a normal distribution with homogeneous data. As demonstrated in Fig. 11, there was a statistically significant difference between sham and MI groups (P-value < 0.05) for all sections.

#### Radial strain for cine MRI

**Figure 12:**
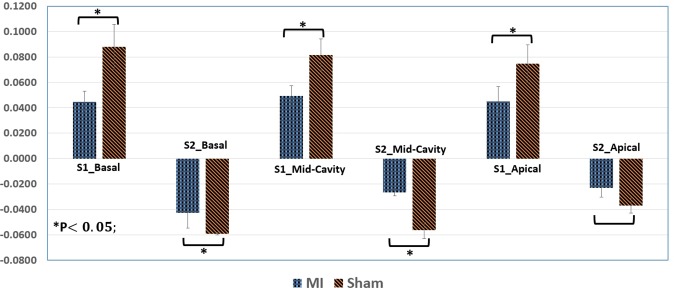
Bar plot for the mean and standard error for mean (SEM) of systolic (S1) and diastolic (S2) for radial strain that estimated for all three cross-sections (basal, mid-cavity, apical) to sham and myocardial infarction (MI) groups. The p-values were obtained based on the Student’s t-test.

Besides the circumferential strain, the radial strain for the inner and outer walls was estimated (inner radial strain between inner and mid walls or outer radial strain between mid and outer walls). These radial strain results are shown in Fig. 4 (supplementary) for two subjects at different cross-sections. There was a high level of variation between the inner and outer radial strain. The value of the outer strain is approximately zero over all the frames and the inner strain curves were usually convex. The strain for the outer wall had a higher range compared with the inner wall. This is clearly manifested with the synchronization between the timing of occurrence of end systole of the ventricular volume curve and the timing of minimal circumferential strain and peak for radial strains. Besides strain curves, Fig. 12 displays the bar plot for the mean and SEM of S1 and S2 for all cross-sections (basal, mid-cavity, apical) for sham and MI groups. The statistical analysis was performed similar to the circumferential strain method outlined in Section 2.4.1., which also revealed a statistically significant difference between sham and MI groups (P-value < 0.05) for all sections except the S2 apical, which clearly has the same trend as others.

#### Sector strain

In addition to circumferential and radial strain curves, local strain can be estimated in each of the 17 segments demonstrated in Fig. 5. Strain for 16-sectors at inner wall for a sham and MI subject is illustrated in Fig. 5 (supplementary). The basal and mid-cavity level contain six curves, with a curve for each sector, and the apical level contains four curves. The strain at the 16 sectors for outer wall is shown in Fig. 6 (supplementary).

#### Validation for strain estimation

For the validation of our developed methods for strain estimation, two validation experiments were conducted using real and synthetic data. The strain estimation from cine MRI has been validated using synthetic phantoms images. The phantom model depends on the physiological features and the LV response during the cardiac cycle. To describe the LV motion, a geometric transformation of the LV that includes translation, torsion, shearing, rotation, and compression was used. We use this transformation to map each location defined in the LV model to correspond spatial point at a certain time instant^37,38^. Furthermore, this transformation was used to generate an inverse motion map that is estimated analytically. This was used to establish correspondences between two points at any two-time instants.

Figure 7 in the supplementary section represents the estimated strain for the mid-wall, from the proposed method in comparison to the GT strain. This figure illustrates the comparison for both original (raw data) and the normalized strain values. The visual comparison shows the good agreement and similar trends between the strain curves, which are also confirmed by the CorCoef and the MSE statistical metrics. The high correlation (CorCoef = 0.89) between the estimated strain points and the ground truth points and low MSE (2.7%) validates the advantage of the proposed methodology.

The strain estimation from tagged MRI has also been validated. In order to validate our method of strain estimation from cardiac tagged images, we used tagged MRI data from three mice subjects. An expert manually tracked myocardial points in all image frames over the cardiac cycle for the tagged MRIs of the subjects. The strain curve from the manually-tracked points was then constructed to form the ground truth strain. Then, the proposed tracking method was applied using the expert selected points on the first image frame and tracked over the rest of the frames during the cardiac cycle. The tracked points were used to construct the strain curves. Quantitative comparison between the estimated strain curves from our method and the ground truth was performed. The MSE was 15e-4 and the CorCoef was 0.941. Also, Fig. 8 in the supplementary materials shows visual comparison between strain curves for a mouse subject.

## Discussion

An accurate, automated algorithm for the assessment of global LV function and structure, as well as the localized strain analysis using cine imaging in mice, was developed. In addition, experiments show the potential of our algorithm to effectively work on mouse hearts, in spite of their tiny sizes. All the recent studies used cine MRI for calculating strain from humans such as Tommaso Mans *et al*.^39^ who estimated 3D myocardium strain from cine MRI using the diffeomorphic demons, a non-linear registration algorithm, by inserting two physical constraints. Myocardium near incompressibility is ensured by constraining the deformations to be divergence-free. Also, myocardium elasticity is modeled using smooth vector filters. Furthermore, Hao Gao *et al*.^40^ estimated myocardial strain from cine MRI using a b-spline deformable image registration method. The automated algorithm enables the use of a single imaging modality to determine both LV structure, strain, and both global and regional function. The proposed algorithm reduces the use of MRI scanning time and cost. It also leads to more reliable estimation of myocardial indices. The novel algorithms presented in the current manuscript provide a means to reliably analyze strain in the mouse heart. This application could conceivably be extended to myocardial strain in humans. Previous studies compared the quality of the data from cine and tagged MRI in human hearts^41^. They demonstrated that both methods are accurate and reproducible and can be used to determine the prognosis and the effect of therapeutic interventions in heart failure patients. Significantly, this algorithm is the first unbiased high-throughput method to analyze cardiac MRI independent of the human bias.

FCN architecture is a deep learning model that is efficient in segmenting different anatomical structures. Here, we demonstrated a successful application of FCN in segmenting the LV cavity and myocardium of mice in both sham and MI hearts. As shown in Table 1, our approach provided accurate cardiac segmentation in terms of DSC and HD, which are widely used measures for segmentation quality. Furthermore, as presented in Fig. 6, our approach performed well at the different LV slices, including the apical slices where it is more difficult to segment the LV as it occupies a small region of the image. The cardiac tissue represents a small proportion in most of the cine MRI images, which makes the network biased towards the majority class (background tissues). Therefore, we implemented a novel loss function that considers this imbalance and penalizes the effect of cross-entropy loss that is prone to class-imbalance problem. Our novel loss algorithm demonstrated its potential by providing high quality segmentation. As presented in Table 1 our loss function resulted in better segmentation metrics than BCE loss, where the problem of class-imbalance is more severe, and Tversky loss, which is used to overcome class-imbalance. Besides our loss function, data augmentation is another key factor for the performance of our approach. Because data augmentation helped us to train our network with a higher sample size and avoided over-fitting. Most of the recent cardiac image analysis studies use human cine MRI, where the images have higher signal-to-noise (SNR) ratio and better contrast, which eases the process of cardiac segmentation^42^. There have been limited studies applied on mice cardiac MRI^21^ where the task is more challenging due to poor SNR. Therefore, the prime merit of our study is the application of deep learning on mice MRI to provide an accurate, preclinical tool for heart segmentation.

Global and local (regional) indices are important to fully characterize cardiac structure and function. Global indices include EF, SV, and LVM; while local indices include myocardial strain, wall thickness, and thickening. To calculate cardiac function and structure parameters, different algorithms based on the Laplace equation were developed and applied. The solution of the Laplace equation between wall borders enabled us to co-allocate corresponding pairs on the borders to estimate the wall thickness and thickening. Previous work was based on center-line-based or radial-based methods^43^, which are not suitable for noisy images and produce large errors due to the lack of strong edges. These methods also assume a circular symmetry for the heart, which can increase the error. To achieve high accuracy for co-localization of the myocardial points, the Algorithm 1 was developed because it depends on geometric features (see Fig. 4).

Another important local index is myocardial strain, which is usually derived from tagged MRI to assess contractile function. However, tagged-MRI-based methods fail in cases of a large displacement or rapid motion between successive image frames^44^. Because strain is not the only index that quantifies the workings of the heart, other performance indices are needed (e.g., EF, wall thickness) and are derived from cine MRI the goal of our work is to develop a pipeline that is capable of estimating the strain and other cardiac indices from a single cardiac (i.e., cine CMRI). This not only helps to avoid the inter-slice variability, but also to afford better correlated indices for cardiac function. Thus, unlike traditional texture-based tracking methods to estimate functional strain (circumferential and radial), we again developed a method that is based on the Laplace equation. Namely, the tracking of myocardial points over the cardiac cycle between successive frames was based also on the solution of the Laplace equation. The proposed method showed the ability to track the movement and rotation of the heart. Hence, it limits the impact of noise that comes from heart motion and enables accurate strain estimation using the Lagrangian-based approach (see Algorithm 2). The obtained results and statistical analyses, in Figs. 9, 10 and Table 3, documented that cine MRI can obviate the need of tagged MRI for strain estimation. One of the challenges that we would like to address in this manuscript is the ability of our method to assess strain from cine imaging and avoid performing tagged imaging in the small mouse heart. However, most of the strain data are correctly reflected in the first 17 frames and there is high consistency between strain acquired from cine and tagged images.

One of the limitations of the current study is the cohort size (n = 6). Although the number of subjects is small, we were able to demonstrate the ability of our developed methodology to accurately estimate strain and other cardiac functional indices from a single cardiac MR modality. Importantly, for the MI group (n = 3), different degrees of myocardial infarction were intentionally induced to assess the ability of the algorithm to detect the changes in the strain in various degrees of heart failure. Therefore, MI-1 has more sever heart failure and dilatation compared to the other two animals in the MI group (see Fig. 8). Our developed algorithm was able to accurately determine the strain from all animal subjects using the cine images. As a future work, we plan to test and validate the algorithm potential using a larger cohort. Despite these limitations, this study demonstrates the feasibility of accurately estimating myocardial strain indices, which may obviate the need for tagged MR clinically.

## Methods

### Animal experiments

All animal procedures were performed in accordance with the National Institutes of Health Guide for the Care and Use of Laboratory Animals and were approved by the University of Louisville Institutional Animal Care and Use Committee. All surgeries were performed as previously described^10,45–47^. Briefly, 10–12-week-old female C57bl6 mice were subjected to non-reperfused MI. Mice were anesthetized with intra-peritoneal injections of ketamine hydrochloride (50 mg/kg) and sodium pentobarbital (50 mg/kg). Anesthetic depth was monitored throughout the surgery. Mice were orally intubated and ventilated with oxygen. A 7–0 silk suture was passed under the left anterior descending coronary artery (LAD) and tied. Sham-operated mice served as surgical controls and were subjected to the same procedure without ligation of the LAD. Mice were extubated upon recovery of spontaneous breathing. Analgesia (ketoprofen, 5 mg/kg) was provided prior to recovery and by 24 and 48 h post-surgery. At the end of the experiments (2 weeks after MI), animals were subjected to magnetic resonance imaging (MRI) to assess cardiac structure and function as described in more detail below.

### Magnetic resonance imaging (MRI)

Our data sets consist of cross-sections cine MRI and tagged MRI for six subjects. About 17 temporal frames for each cross-section through the cardiac cycle were collected. We obtained about eight cross-sections for the complete coverage of the LV for each subject. These datasets contain three sham and three MI subjects.

All MR imaging data were acquired using Agilent 9.4*T* horizontal bore MRI system equipped with Agilent 205/120 HD gradient coil (Agilent Technologies, Santa Clara, CA, USA). A RAPID 72-mm volume coil was used for signal transmission and a 4-channel mouse heart surface coil was used for signal detection (RAPID MR International, Columbus, OH, USA). Surface coil was positioned below the mouse body. During *in vivo* MRI scans, mice were anesthetized with 1.5% isoflurane in 100%*O*_2_. Body temperature, breathing rate as well as Electrocardiography (ECG) signals were continuously monitored using a small animal monitoring and gating system (Model 1030, SA Instruments, Inc., Stony Brook, NY, USA). All cine and tagged MRI scans were gated with both ECG and breathing.

After two quick heart scout scans to obtain long axis 4-chamber and 2-chamber view images, high resolution short axis view cine images were obtained using the following parameters: *TR*/*TE* = 5.0/1.6 msec; flip angle =15° matrix size = 30 × 30 *mm*^2^; field of view (FOV) = 256 × 256 *mm*^2^; 8 slices with a slice thickness of 1.0*mm*; number of averages = 2. As for black blood tagged cine, the scanning parameters were: TR/TE = 5.0/1.9 msec; flip angle =20° matrix size = 30 × 30 *mm*^2^; FOV = 128 × 128 *mm*^2^; 8 slices with a slice thickness of 1.0*mm*; number of averages = 4; tagging resolution =0.3*mm* with 0.6*mm* separation; tag time = 16.24 msec.

## Conclusion

In conclusion, we have developed a new automated algorithm to accurately assess cardiac function and strain parameters from cardiac cine MR images. This new algorithm may void the need for cardiac tagged MR imaging which is time consuming and hard to achieve in rodents.

## Supplementary information

Supplementary Materials.

## Data Availability

Materials, data, and associated protocols will be available to readers after the manuscript being accepted.
