# Supplementary material for: A New Framework for Performing Cardiac Strain Analysis from Cine MRI Imaging in Mice
Source: Sci Rep. 2020 May 7;10:7725. doi: 10.1038/s41598-020-64206-x (PMC7205890; doi:10.1038/s41598-020-64206-x)
Supplement: Supplementary file 1 — Supplementary Materials. [file 41598_2020_64206_MOESM1_ESM.pdf]

A New Framework for Performing Cardiac Strain Analysis from Cine MRI Imaging in Mice: Supplementary

K. Hammouda[1,*], F. Khalifa[1,*], H. Abdeltawab[1,*], A. Elnakib[1], G. A. Giridharan[1], M. Zhu[2], C. K. Ng[2], S. Dassanayaka[3], M. Kong[4], H. E. Darwish[5], T. M. A. Mohamed[3, 6, +], S. P. Jones[3, +], and A. El-Baz[1, +, x]

[1]BioImaging Laboratory, Department of Bioengineering, University of Louisville, Louisville, KY, USA
[2]Electronics and Communications Engineering Department, Faculty of Engineeering, Mansoura University, Egypt
[3]Department of Radiology, Department of Medicine, University of Louisville, Louisville, KY, USA
[4]Diabetes and Obesity Center, Department of Medicine, University of Louisville, Louisville, KY, USA
[5]Department of Bioinformatics and Biostatistics, SPHIS, University of Louisville, Louisville, KY, USA
[6]Mathematics Department, Faculty of Science, Mansoura University, Mansoura, Egypt
[7]Division of Cardiovascular Medicine, Department of Medicine, University of Louisville, Louisville, KY, USA
[*]These authors contributed equally to this work
[+]These authors share senior authorship
[x]Correspondence to aselba01@louisville.edu

ABSTRACT

Cardiac magnetic resonance (MR) imaging is one of the most rigorous form of imaging to assess cardiac function *in vivo*. Strain analysis allows comprehensive assessment of diastolic myocardial function, which is not indicated by measuring systolic functional parameters using with a normal cine imaging module. Due to the small heart size in mice, it is not possible to perform proper tagged imaging to assess strain. Here, we developed a novel deep learning approach for automated quantification of strain from cardiac cine MR images. Our framework starts by an accurate localization of the LV blood pool center-point using a fully convolutional neural network (FCN) architecture. Then, a region of interest (ROI) that contains the LV is extracted from all heart sections. The extracted ROIs are used for the segmentation of the LV cavity and myocardium via a novel FCN architecture. For strain analysis, we developed a Laplace-based approach to track the LV wall points by solving the Laplace equation between the LV contours of each two successive image frames over the cardiac cycle. Following tracking, the strain estimation is performed using the Lagrangian-based approach. This new automated system for strain analysis was validated by comparing the outcome of these analysis with the tagged MR images from the same mice. There were no significant differences between the strain data obtained from our algorithm using cine compared to tagged MR imaging. Furthermore, we demonstrated that our new algorithm can determine the strain differences between normal and diseased hearts.

Keywords: Cardiac MR, Cardiac Parameters, Deep Learning, Left Ventricle, Segmentation.

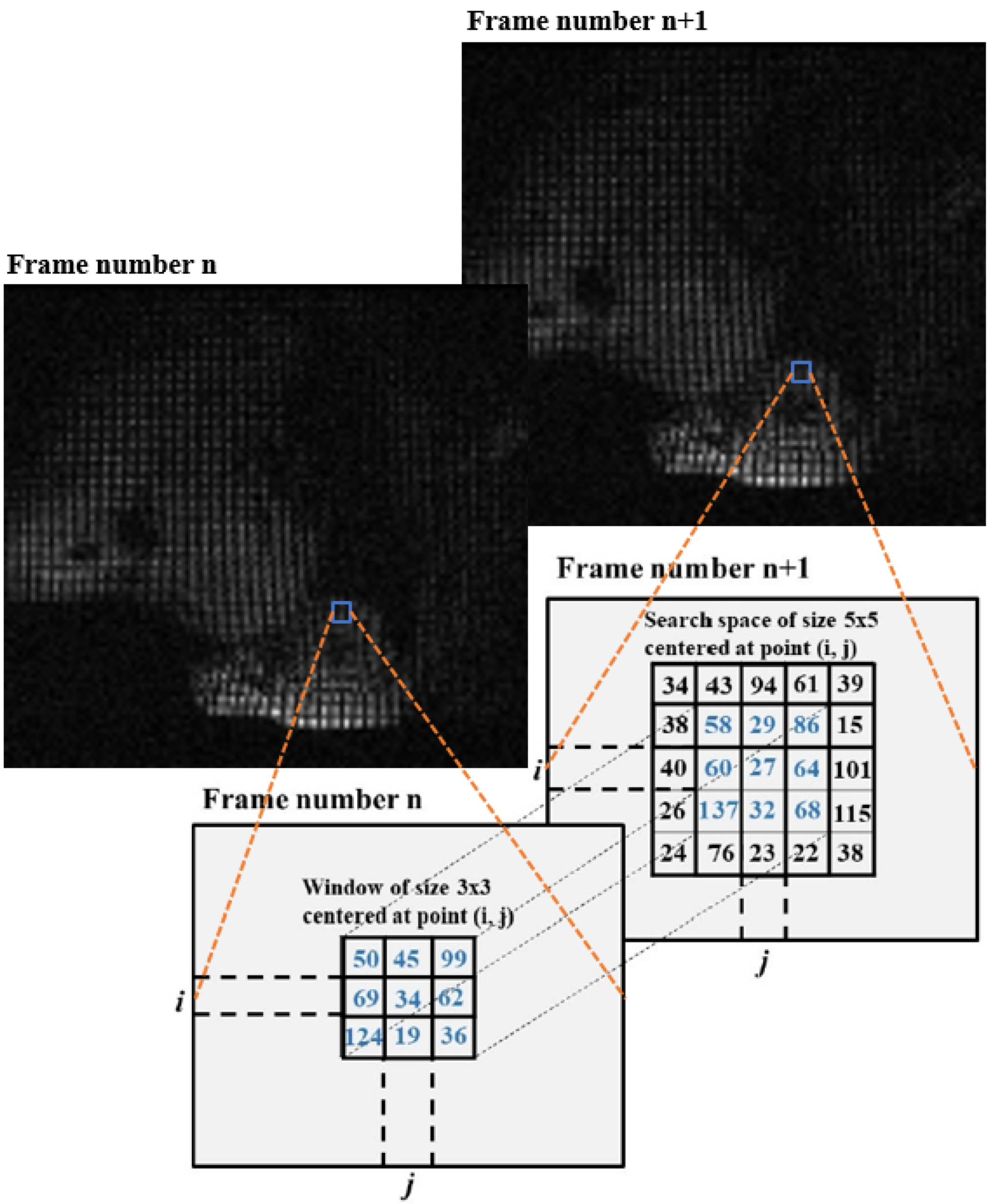

Figure 1. Tracking of tagged MRI pixel (i, j) in frame number n by finding the maximum correlation between a window of size 3x3 and a window of size 5x5 in frame number $n+1$.

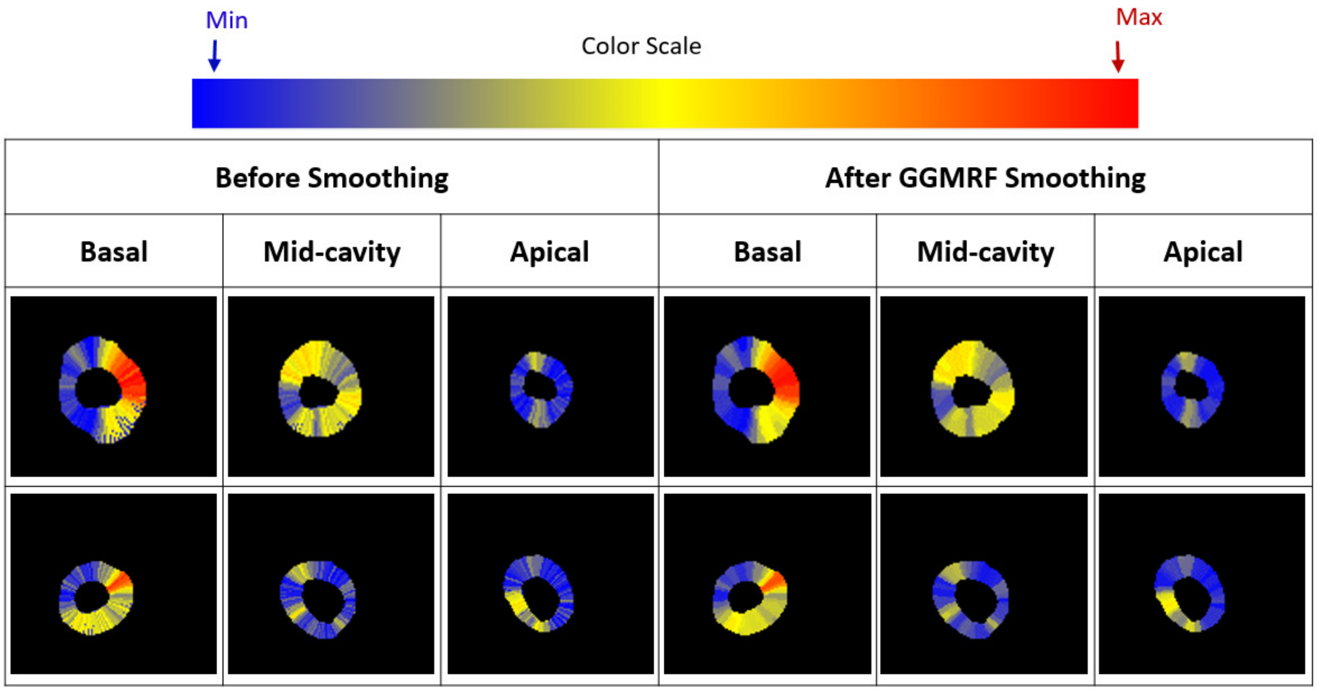

Figure 2. The color-map representation of the wall thickening for one subject, the first row is a sham subject and the second row is a myocardial infarction (MI) subject. The red and blue hues of the color scale relate to the maximum and minimum thickening, respectively. For continuity, the estimated maps are modeled using Gauss-Markov random field (GGMRF) model.

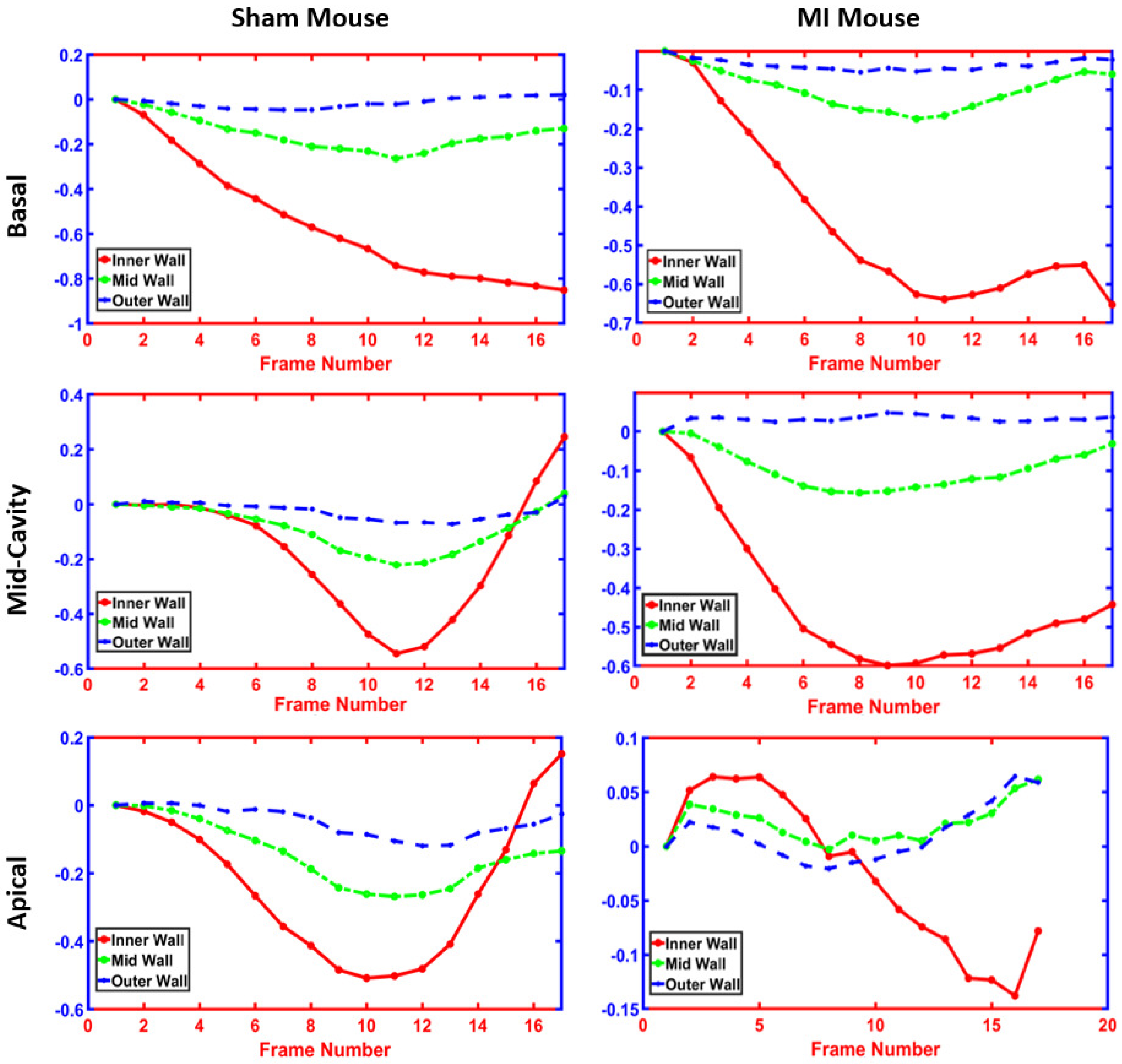

Figure 3. Estimated circumferential strain for inner, mid and outer walls of the LV at different cross-sections (slices) for a sham (left) and a myocardial infarction (MI) (right) subject.

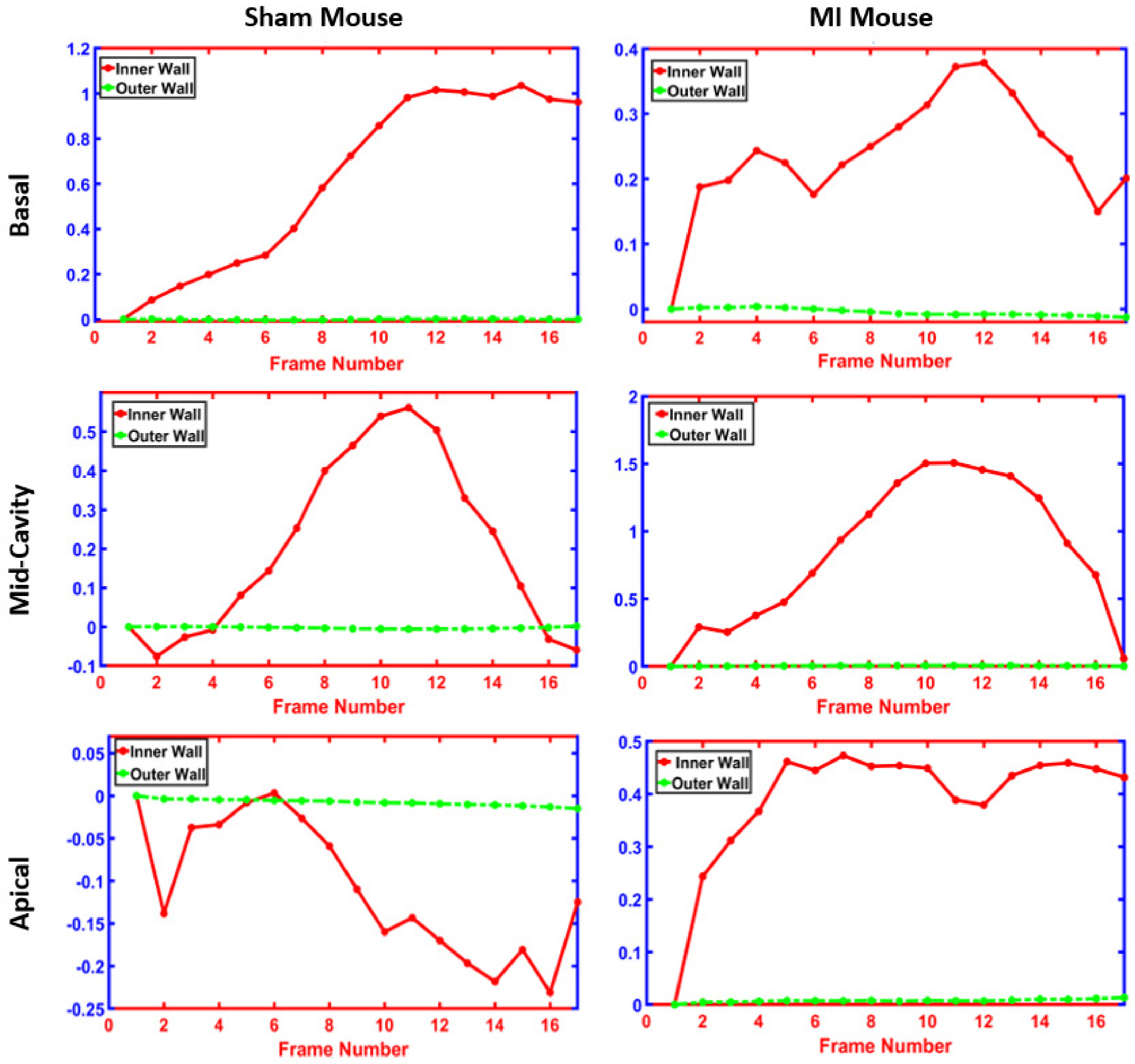

Figure 4. Estimated radial strain for inner and outer walls of the LV at different cross-sections (slices) for a sham (left) and a myocardial infarction (MI) (right) subject.

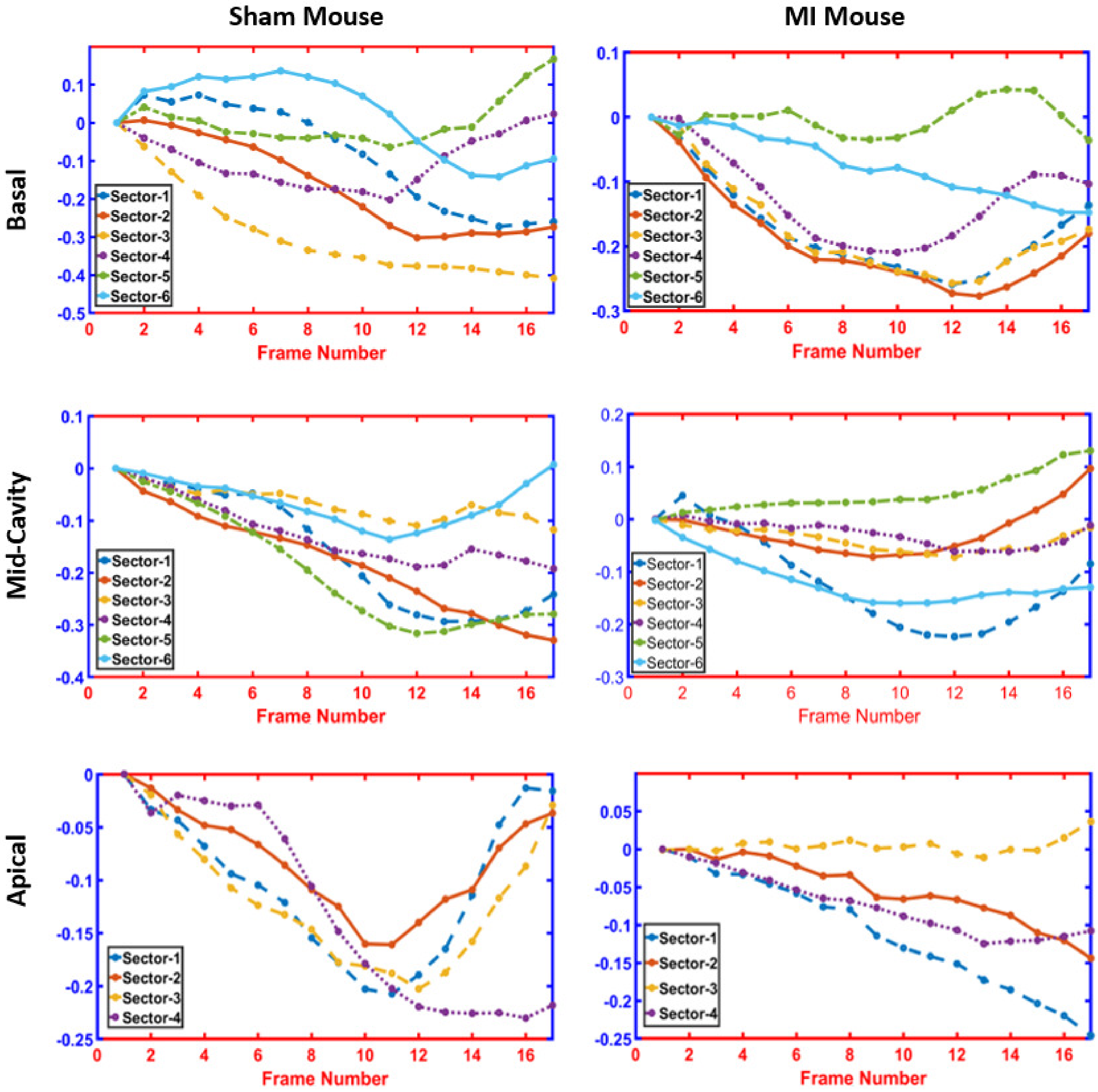

Figure 5. Examples of sector-strains estimated at inner wall for sham and myocardial infarction (MI) subjects.

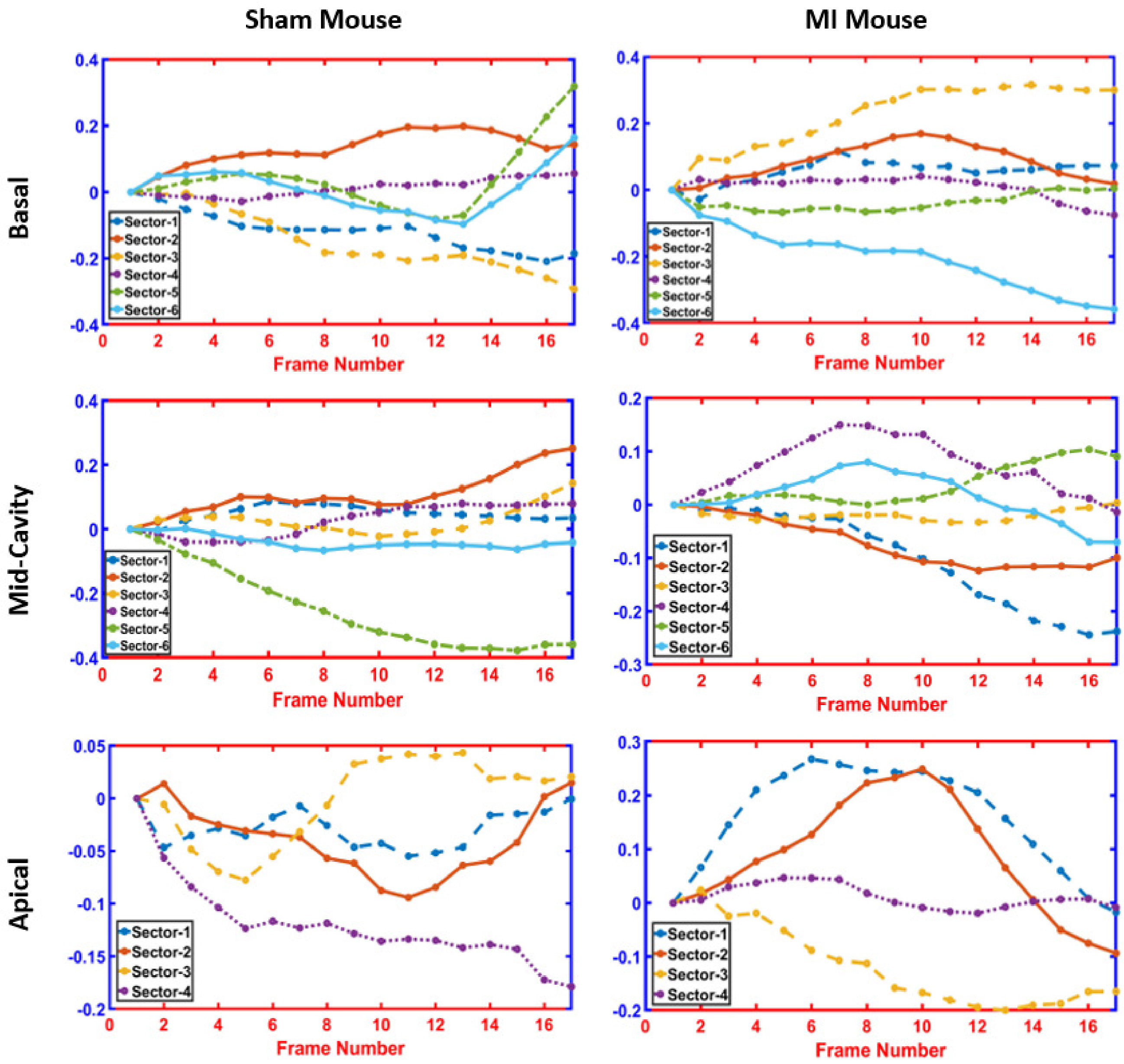

Figure 6. Examples of sector-strains estimated at outer wall for sham and myocardial infarction (MI) subjects.

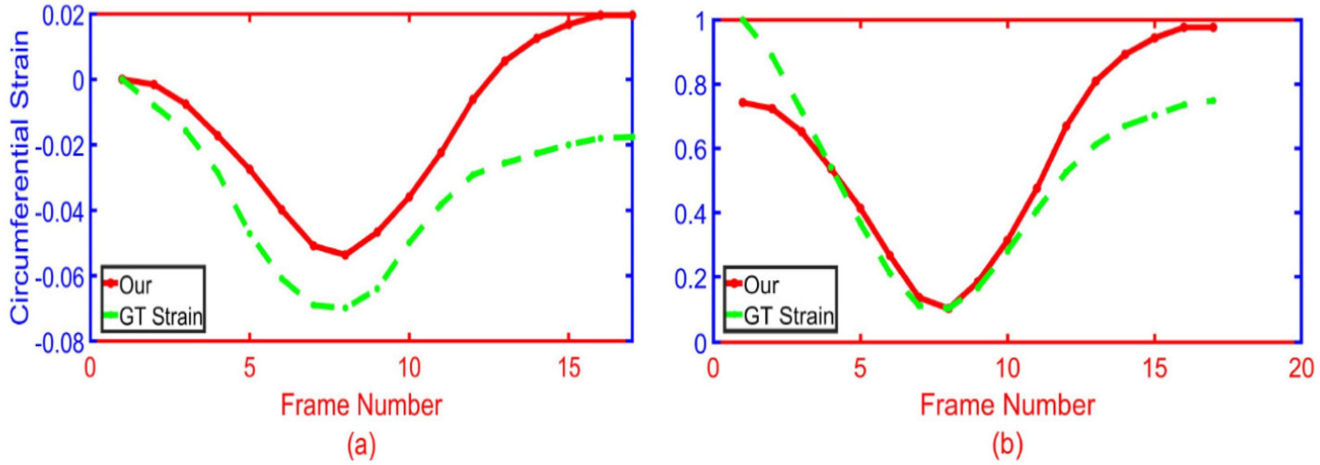

Figure 7. Comparison results between our strain estimation and the GT strains (obtained from the deformation of the phantom model), (a) shows the comparison with the original values for the strain (raw data) and (b) the comparison with the normalized values, between 0 and 1 (b).

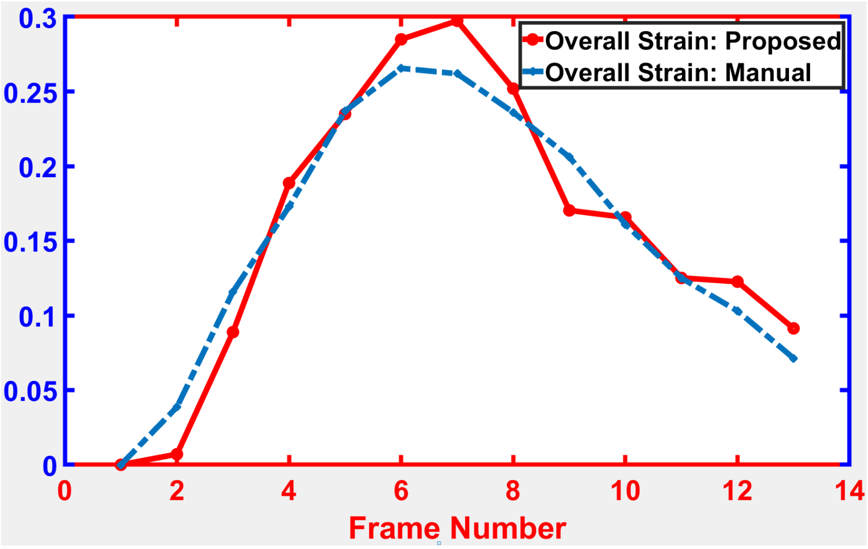

Figure 8. Comparison between the constructed strain curve from both the manual tracking by an MRI expert and the automated tracking by our proposed method.
